# Naringin attenuates inflammatory injury to the bovine endometrium by regulating the endoplasmic reticulum stress–PI3K/AKT–autophagy axis

**DOI:** 10.3389/fphar.2024.1424511

**Published:** 2024-08-21

**Authors:** Zihao Lu, Qingyang Peng, Ruiting Hu, Yan Wang, Kewei Fan, Tao Zhang

**Affiliations:** ^1^ College of Animal Science and Technology, Anhui Agricultural University, Hefei, China; ^2^ Longyan University and Fujian Provincial Key Laboratory for Prevention and Control of Animal Infectious Diseases and Biotechnology, Longyan, China

**Keywords:** naringin, endometritis, ERS, autophagy, network pharmacological

## Abstract

**Background:** Endometritis seriously affects maternal reproductive health and fertility. Natural compounds have the characteristics of high efficiency and low residue in disease treatment. We aimed to discover and reveal the pharmacological effects of naringin, which is widely present in food and plants, on endometritis.

**Methods:** Based on network pharmacology, the potential targets and pathways of naringin’s actions on endometritis were predicted. Animal *in vivo* experiments were conducted to examine the inflammatory response of lipopolysaccharides (LPSs) in uterine tissue and the therapeutic effect of naringin. An *in vitro* primary bovine endometrial epithelial cell inflammation and drug treatment model was constructed. The production of reactive oxygen species (ROS) was measured using DCFH-DA, and the effect of naringin on LPS-induced endometritis was evaluated using HE staining, real-time quantitative PCR, Western blot, and immunofluorescence staining methods.

**Results:** Naringin alleviated LPS-induced inflammatory injury and oxidative stress in the endometrium of mice and bovine endometrial epithelial cells (bEECs). Furthermore, *in vitro* studies were carried out to reveal the potential anti-inflammatory mechanisms of naringin based on network pharmacology. We found that naringin significantly inhibited LPS-stimulated endoplasmic reticulum stress (ERS)-related gene and protein expression, thus reducing the unfolded protein response (UPR). Furthermore, treatment of naringin attenuated the autophagic flux induced by ERS. In a further study, we observed that PI3K/AKT pathway inhibitors or ERS inducers partially reverse naringin’s inhibition of autophagy and cell apoptosis.

**Conclusion:** It is demonstrated that naringin suppresses autophagy by directly inhibiting the ERS-PI3K/AKT axis and exerting anti-inflammatory and antioxidant effects in endometritis. These findings provide novel insights into the pathogenesis of endometritis, highlighting potential therapeutic targets of traditional herbs and compounds.

## Graphical Abstract

**Figure:**
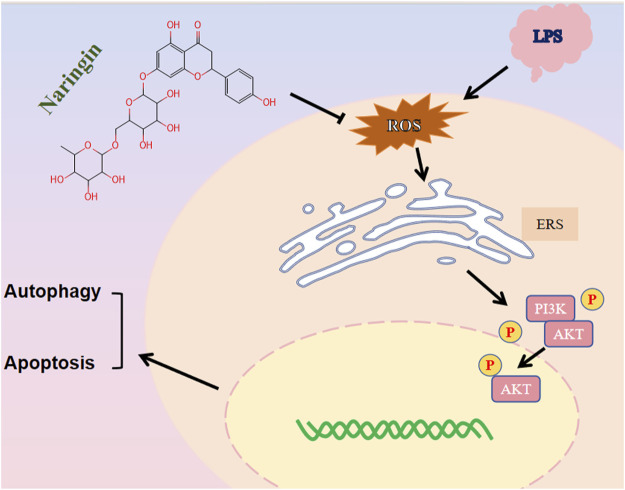
Naringin activates the PI3K/AKT pathway by reducing ROS production.

## 1 Introduction

The integrity of the endometrium structure and function prepares the bovine uterus for embryo implantation and pregnancy, and it is also related to women’s health. Previous studies have shown that bovine uterine inflammation is related to local disturbances in the metabolism of amino acids, lipids, and carbohydrates in the uterus (Figueiredo et al., 2023). However, the mechanisms by which the cells of the endometrium respond to infection remain unclear, and existing treatment strategies have a poor prognosis or bring side effects such as drug resistance.

Naringin (4’,5,7-trihydroxyflavonone-7-rhamnoglucoside) is an important flavonoid existing in the fruit or peel of rutaceous grapefruit, which contributes to the bitter taste of citrus juices (Dong et al., 2023). Accumulating evidence suggests that naringin possesses a variety of pharmacological activities, including antioxidant, anti-inflammatory, and antiapoptotic effects (El-Desoky et al., 2018; Raghu et al., 2023). Naringin has also been proven to have the advantages of high safety, few side effects, and no antibiotic residue in the treatment of diseases, like other traditional Chinese medicines (Jiang et al., 2021; Xia et al., 2023). Due to the multi-target nature of traditional Chinese medicine, the specific molecular mechanisms by which naringin functions need to be explored. For example, DSS induced the activation of the MAPK pathway, and the NLRP3 inflammasome was inhibited by naringin (Cao et al., 2018). Naringin induced autophagy cell death (Raha et al., 2020). Recent studies also showed that naringin induced cancer cell apoptosis by repressing the non-coding RNAs or the PI3K/AKT signaling pathway (Nie et al., 2012). However, the effects and potential mechanisms of naringin on bovine endometritis are still unknown.

The endoplasmic reticulum (ER) is an important organelle in eukaryotic cells and is responsible for protein synthesis, lipid formation, Ca^2+^ storage, and signaling (Díaz-Villanueva et al., 2015; Christianson et al., 2023). ER stress (ERS) is a cellular response to various stimuli, such as protein misfolding, hypoxia, the change in Ca^2+^ levels, and mutant proteins, that disrupt the normal function of the ER (Marciniak et al., 2022). On the one hand, ERS triggers the unfolded protein response (UPR) as a therapeutic strategy to repair ER homeostasis by promoting ER-associated degradation (ERAD) and clearing unfolded proteins, reducing protein synthesis and increasing protein folding capacity (Hetz et al., 2020; Marciniak et al., 2022). On the other hand, prolonged ERS can inhibit autophagy, promote cell apoptosis, and participate in the pathogenesis of various diseases, such as neurodegeneration, cancer, and inflammation (Li et al., 2023). Meanwhile, studies have reported a close relationship between autophagy and the ERS pathway or that the two affect each other (Díaz-Villanueva et al., 2015; Hetz et al., 2020). However, in some cell types (such as tumor cells), autophagy leads to cell death when protein and organelle turnover exceeds the threshold of the cells (Kumariya et al., 2021). Many studies have shown ERS leads to the induction of autophagy, and organelle autophagy is considered a potential drug target (Mochida and Nakatogawa, 2022; Christianson et al., 2023). Currently, it is not clear whether naringin can treat endometritis through ERS and autophagy.

Herein, we aimed to investigate the effect of naringin on lipopolysaccharide (LPS)-induced uterine inflammatory injury for a better understanding of the potential mechanism of naringin treatment on endometritis. We focus on its ability to regulate the ERS–autophagic system and apoptotic processes. The current study is important for screening novel treatment strategies and potential drug targets for endometritis.

## 2 Materials and methods

### 2.1 Reagents and antibodies

Naringin (purity ≥98%) was obtained from Shanghai Yuanye Bio-Technology Co., Ltd. (Shanghai, China). 3-MA, LY294002, AKT inhibitor IV, and tunicamycin (TM) were purchased from MedChemExpress. LPS (*E. coli* 055:B5) was purchased from Sigma-Aldrich. Anti-PERK (#AF5304), anti-p-PERK (#DF7576), anti-IRE1α (#DF7709), anti-p-IRE1α(#AF7150), anti-ATF6 (#DF6009), anti-Bcl-2 (#AF6139), anti-PI3K (#AF6241), anti-p-PI3K (#AF3241), anti-AKT (#AF6261), anti-p-AKT (#AF0016), and anti-β-actin (#AF7018) were purchased from Affinity. Anti-LC3 A/B (#12741), anti-p62 (#23214), and anti-Beclin-1 (#3495) were purchased from Cell Signaling Technology. Anti-Bax (ab32503), anti-cleaved caspase-3 (ab214430), goat anti-rabbit (ab6721), and anti-mouse (ab6789) secondary antibodies were purchased from Abcam.

### 2.2 Network pharmacological analysis

The chemical structure and simplified molecular input line entry specification (SMILES) of naringin were obtained from TCMSP (https://www.tcmsp-e.com/tcmsp.php) and the PubChem website (https://pubchem.ncbi.nlm.nih.gov/). Target prediction of naringin was carried out using the TCMSP, the SwissTarget Prediction database (http://www.swisstargetprediction.ch), the STITCH database (http://stitch.embl.de/), and PharmMapper (http://www.lilabecust.cn/pharmmapper/), while merging the target targets of naringin obtained from the above three databases and remove duplicate and non-standard targets. Using the keywords “uterus and inflammation or endometritis,” targets related to inflammatory injury of uterine were searched for and collected through the DrugBank (http://www.drugbank.com), GeneCards (https://www.genecards.org/), OMIM (https://omim.org/), NCBI Gene (https://www.ncbi.nlm.nih.gov/gene), and DisGeNET (https://www.disgenet.org/) databases. The screening criteria for targets related to endometritis were the target of action that appears simultaneously in two or more databases using the Venny online tool. Intersected target genes from naringin and endometritis were uploaded to DAVID (http://david.abcc.ncifcrf.gov/) to determine functional term enrichment, including gene ontology (GO) enrichment and Kyoto Encyclopedia of Genes and Genomes (KEGG) pathway analysis. GO enrichment analysis and KEGG pathway analysis were performed using the bioinformatics platform (http://www.bioinformatics.com.cn/).

### 2.3 Animals and treatment

All mice were maintained on a 12-h light/12-h dark cycle with free access to food and water. The animals involved in this research are raised and managed in strict accordance with the Regulations on the Management of Experimental Animals and the guidelines formulated by the Experimental Animal Ethics Committee of Anhui Agricultural University, and all animal experiments are conducted in strict accordance with the norms of the Ethics Committee of Anhui Agricultural University.

Female Kunming mice (6 weeks old) were provided by the Laboratory Animal Center of Anhui Medical University (Hefei), product license number SCXK (Anhui) 2017–001. Intrauterine LPS was injected at a concentration of 0.5 mg/kg to create the endometritis model, and a blank control group was given the same dose of PBS solution (Zhang et al., 2022). At the same time, naringin (100 μL) was given by oral gavage at concentrations of 20 mg/kg, 40 mg/kg, and 80 mg/kg. For autophagy inhibitors, PI3K/AKT inhibitors, or ERS inducer treatment, mice received 3-MA (10 mg/kg) and LY294002 (25 mg/kg) by intraperitoneal injection at 30 μL 12 h after treatment with naringin. TM (2.5 mg/kg, 30 μL) was given by oral gavage. The mice were euthanized 24 h after naringin treatment, and the uterine tissue was collected for analysis. Each experimental and control group had 12 mice.

### 2.4 Cell culture and treatment

Primary bovine endometrial epithelial cells (bEECs) were isolated and cultured following the procedure documented in our previous study (Zhang et al., 2021). To investigate the mechanism of naringin on LPS-induced bovine endometritis, LPS (1ug/mL) was used to treat epithelial cells to simulate inflammation *in vitro* (Zhang et al., 2023). bEECs (60% confluent) were exposed to 25 μM, 50 μM, and 100 μM naringin for 24 h, and then cells were collected and stored at −20°C for the next experiment. LY294002 (10 μM), AKT inhibitors IV (2 μM), and TM (5 ug/mL) were administered 2 h prior to naringin treatment. Equal amounts of PBS or DMSO were used as a negative control group. After the specified treatment, the cells were prepared for further experiments.

### 2.5 Cell viability and cytotoxicity assay

Cell viability was measured using CCK-8 assay (Dojindo Laboratories, Tokyo, Japan) according to the manufacturer’s instructions. In brief, cells were seeded in 96-well plates at a density of 3 × 10^4^ cells/mL, with five repetitions for each group. After treatment, cells were continuously cultured with 10 μL of CCK-8 in each well at 37°C for 2 h. The absorbance was determined at 450 nm using a microplate reader (Bio-Rad Instruments, Hercules, CA).

### 2.6 Reactive oxygen species (ROS) assay

As described previously, DCFH-DA (Beyotime, Shanghai, China) could be used to measure ROS production. Cells were seeded at a density of 1 × 10^5^ cells mL^−1^ into 6-well plates. Next, they were incubated with control media (PBS) or LPS (1 μg/mL) in the presence or absence of naringin (25 μM, 50 μM, and 100 μM) for 24 h. The cells were incubated with 2′,7′- dichlorofluorescein diacetate (DCFH-DA, 10 mM) for 30 min at 37°C in the dark and then washed three times with PBS to remove extracellular DCFH-DA. After that, the relative levels of fluorescence were quantified by using a fluorescence plate reader (485 nm excitation and 535 nm emission, Olympus) or a flow cytometer (BD Biosciences, San Jose, California, United States), and FlowJo10 was used to analyze the data.

### 2.7 Histological analysis

Tissue samples were fixed overnight in 4% paraformaldehyde, embedded in paraffin, and then sliced into 4-µm-thick sections. The sections were stained with hematoxylin and eosin (H&E), and the histopathological changes were subsequently examined under a light microscope.

### 2.8 Myeloperoxidase (MPO) assessment

The tissue was accurately weighed, and a 1:19 weight preparation of 5% tissue homogenate (tissue homogenate mixed as much as possible) was prepared. The MPO activity in the sample was detected with a kit (Nanjing Institute of Jianguo Bioengineering, Nanjing, China), and the absorbance peak (optical density, OD) was measured at the wavelength of 460 nm using an enzyme marker. Absorbance was defined as MPO activity = (OD determination −OD control)/(11.3× weight).

### 2.9 Quantitative real-time PCR

TRIzol reagent (Yeasen) was used to extract total RNA. A cDNA Synthesis Kit (Vazyme) was used to reverse transcribe 1 µg of total RNA into cDNA. The primer sequences were designed based on GenBank cDNA sequences. Levels of GAPDH (internal control), IL-6, IL-10, IL-1, and TNF-αmRNAs were analyzed using the SYBR RT-PCR Kit (Takara, Dalian, China). Quantitative PCR (qPCR) was performed using the MiniOpticon qPCR detection system (Bio-Rad Laboratories). The 2^−ΔΔCT^ method was adopted to calculate the relative quantification. Primer sequences are shown in Table 1.

**TABLE 1:** Oligonucleotide primers used for qPCR.

IL-6	Forward	CTG​GTC​TTC​TGG​AGT​ACC​ATA​GC
	Reverse	CTG​GTC​TTC​TGG​AGT​ACC​ATA​GC
IL-1β	Forward	GCC​ACC​TTT​TGA​CAG​TGA​TGA​G
	Reverse	GCC​ACC​TTT​TGA​CAG​TGA​TGA​G
TNF-α	Forward	GAT​CGG​TCC​CCA​AAG​GGA​TG
	Reverse	CCA​CTT​GGT​GGT​TTG​TGA​GTG
IL-10	Forward	GTA​GAA​GTG​ATG​CCC​CAG​GC
	Reverse	CAC​CTT​GGT​CTT​GGA​GCT​TAT​T
GAPDH	Forward	GGTCACCAGGGCTGCTTT
	Reverse	CTGTGCCGTTGAACTTGC
CHOP	Forward	TCT​GGC​TTG​GCT​TAC​TGA​GG
	Reverse	GAC​TGG​CCA​CTC​TGT​TTC​CG
ATF4	Forward	CTT​CGA​CCA​GTT​GGG​TTT​GG
	Reverse	ATT​CGG​AGG​AGC​CTG​CCT​TA
GRP78	Forward	CCT​GTT​CCG​TTC​CAC​CAT​GA
	Reverse	CTT​TCG​TCA​GGG​GTC​GTT​CA

### 2.10 Western blot

Cells and paired primary tissues were extracted by RIPA lysis buffer with freshly added 1% phosphatase inhibitor, 1% phenylmethanesulfonyl fluoride (PMSF), and 0.1% protease inhibitor. The protein was subsequently separated using a 10%–15% SDS polyacrylamide gel and transferred to the PVDF membrane. These membranes were then blocked using 5% skimmed milk for 1 h. Following this, the membranes were incubated at 4°C overnight with specific primary antibodies. After washing with TBST, the membranes were further incubated with secondary antibodies at room temperature for 2 h. Subsequently, the results were detected by employing ECL detection reagents. It is important to note that each experiment was repeated three times for statistical significance.

### 2.11 Immunofluorescence staining

Cells grown on glass coverslips or uterine tissue sections (4 mm) were fixed with 4% paraformaldehyde for 15 min at room temperature (RT), washed three times with PBS, and then permeabilized with 0.05% Triton X-100 for 10 min at RT. Following three washes of 5 min in PBS, the coverslips were blocked with 5% BSA for 30 min and then incubated overnight with antibodies to LC3, XBP1, ATF6, ATF4, and p-AKT at 4°C. Following three washes of 5 min in PBST, the coverslips were incubated with secondary antibodies for 1 h at RT in the dark. Following three washes of 5 min with PBST, the coverslips were stained with DAPI. Images were obtained using an Imager Nikon Eclipse C1 (Japan), a Nikon DS-U3 (Carl Zeiss), or an optical microscope (Olympus, Japan).

### 2.12 Statistical analysis

The *in vitro* experiments were repeated at least three times unless stated otherwise. As indicated in the figure legends, all quantitative data are presented as the mean ± S.D. or mean ± S.E.M. of three biologically independent experiments or samples. Statistical analyses were performed using GraphPad Prism 8 and Excel. Statistical significance was tested using unpaired two-way ANOVA with Sidak’s multiple-comparisons test. All data were considered statistically significant at *p < 0.05, **p < 0.01, and ***p < 0.001.

## 3 Results

### 3.1 Naringin alleviates LPS-induced uterine inflammatory damage in mice

**FIGURE 1:**
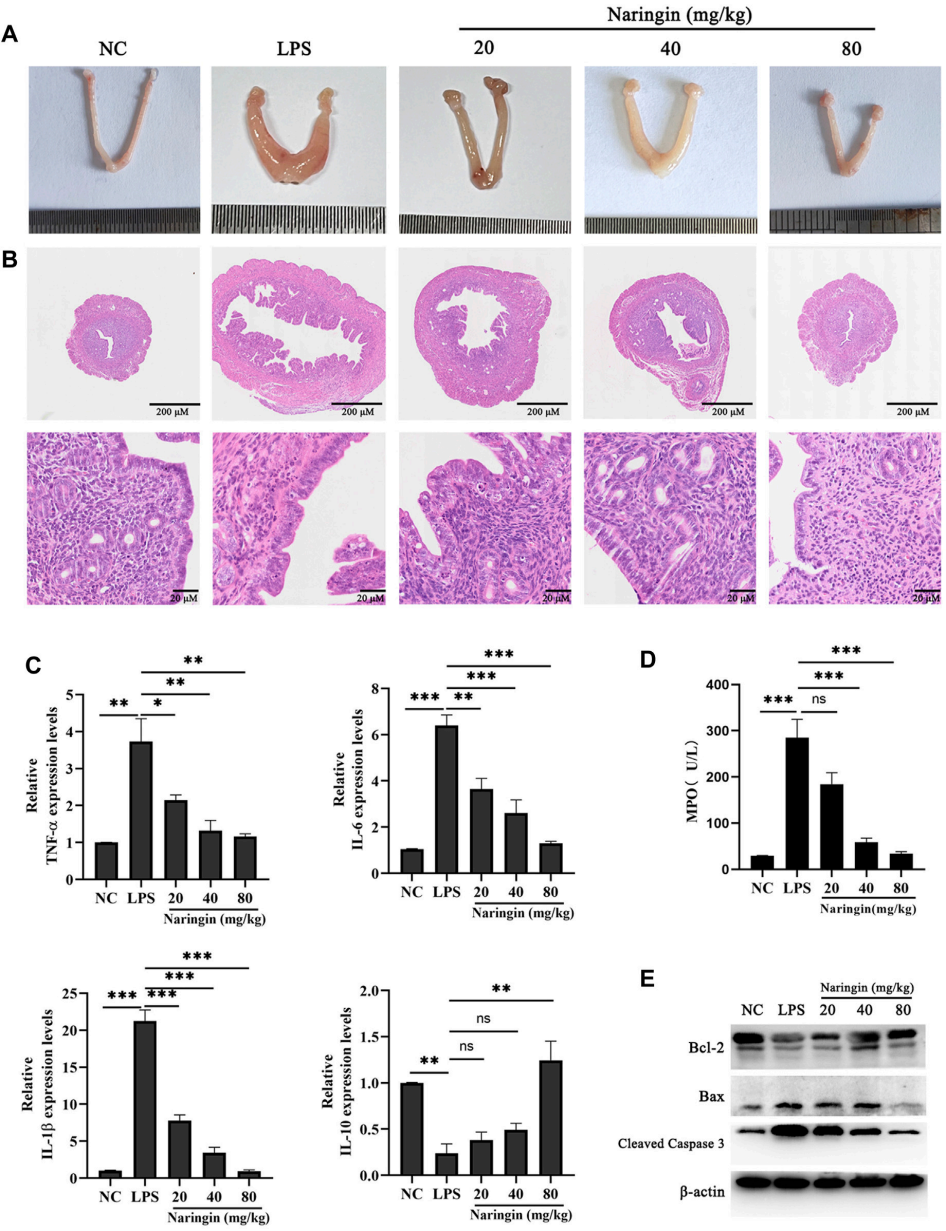
Effects of naringin on LPS-induced uterine injury in mice. The mouse model of endometritis was established by administering different concentrations of naringin (20 μM, 40 μM, and 80 μM) for 24 h, with 12 mice in each group. **(A)** Representative whole uterus images. **(B)** Representative histopathological images of paraffin-embedded mouse uterine sections. Scale bar: 200 μm (top), 20 μm (bottom). n = 5. **(C)** mRNA levels of IL-6, IL-1β, TNF-α, and IL-10 in the endometrium tissues were measured by RT‒qPCR. GAPDH serves as the control. n = 3. **(D)** MPO activity. n = 3. **(E)** Lysates of uterine tissue obtained at different treatments were analyzed for the presence of the indicated proteins. n = 3. All data are represented as the mean ± S.E.M. Experiments were repeated n times with duplicate biological replicates. *p < 0.05; **p < 0.01; ***p < 0.001.

In this study, we analyzed the morphology and histopathological changes in uterine tissues to assay the pharmacological effects of naringin. The results revealed that naringin significantly alleviates LPS-induced pathologic changes in terms of hyperemia, extensive inflammatory cell infiltration, and the structure of the uterus being damaged (Figures 1A, B). As expected, naringin reduced the MPO activity of uterine tissue compared to the control group (Figure 1C). The mRNA levels of the proinflammatory mediators IL-6, IL-1β, and TNF-α and anti-inflammatory mediator IL-10 were analyzed by qPCR. The results showed that the IL-6, IL-1β, and TNF-α were decreased, and the IL-10 was increased in mice with endometritis treated with naringin (Figure 1D). This indicates that naringin can significantly reduce the level of endometritis. In addition, we found that in the presence of naringin, the increases of Bax/Bcl-2 and cleaved caspase-3 induced by LPS were abolished (Figure 1E and attached Supplementary Figure 1). These results indicated that naringin alleviates LPS-induced inflammatory injury in mice.

### 3.2 Effects of naringin administration on the cell viability and inflammation in bEECs

**FIGURE 2:**
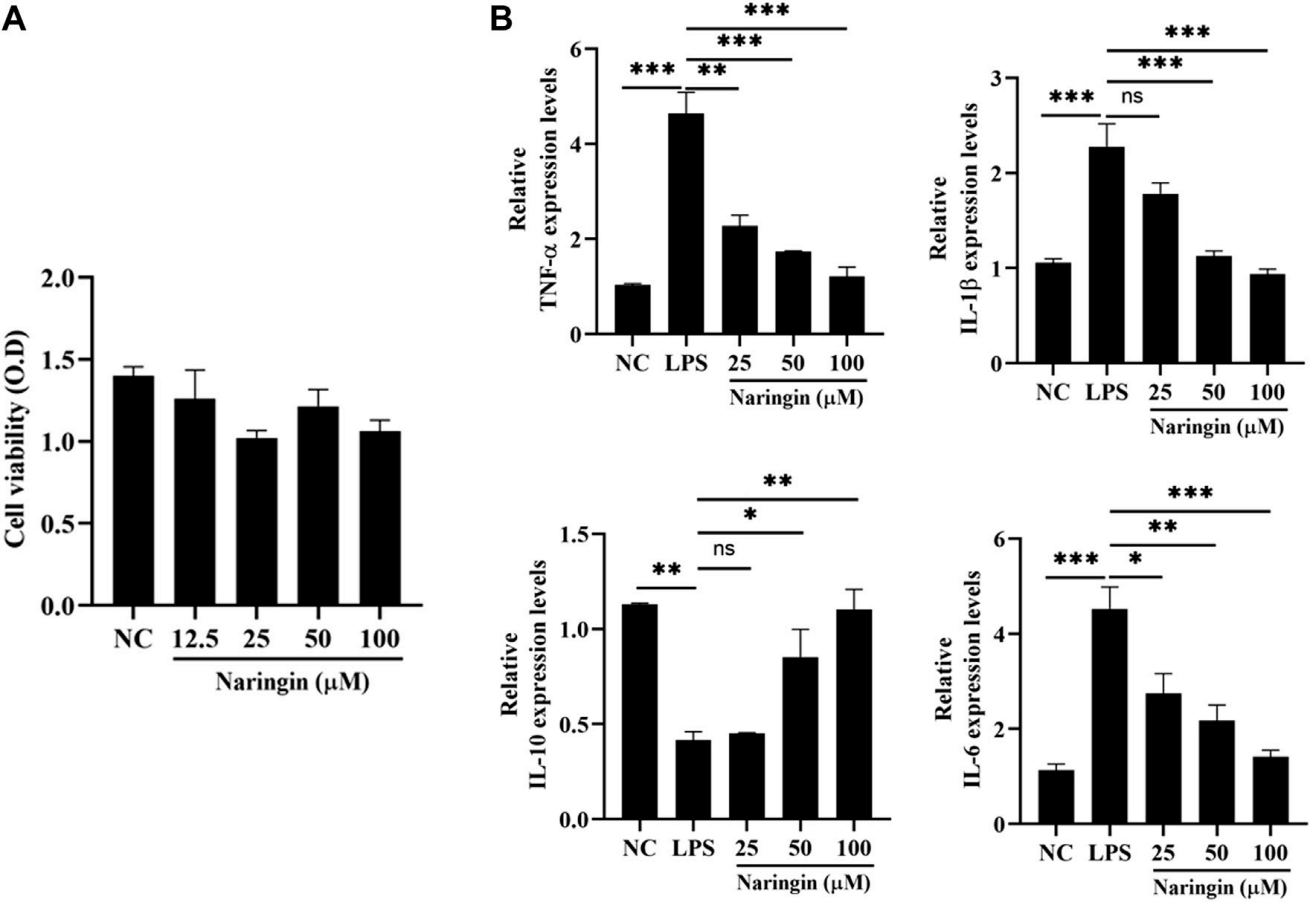
Effects of naringin on LPS-induced inflammatory responses in bEECs. Cells were treated with LPS (1 μg/mL) and then subjected to different concentrations of naringin (25 μM, 50 μM, and 100 μM) and an equal volume of PBS for 24 h. **(A)** Cell viability was measured using a CCK-8 assay. n = 3. **(B)** Expression of IL-6, IL-1β, TNF-α, and IL-10 mRNA in bEECs. GAPDH serves as the control. n = 3. All data are represented as the mean ± S.E.M. Experiments were repeated n times with duplicate biological replicates. *p < 0.05; **p < 0.01; ***p < 0.001.

In order to further investigate the anti-inflammatory mechanism of naringin, we isolated and identified primary bovine endometrial epithelial cells (attached Supplementary Figure 2). The effect of naringin on the viability of bEECs was detected by the CCK-8 kit. The results showed that cell viability was not affected by naringin administration (Figure 2A). Next, we measured the expression of proinflammatory cytokines IL-6, IL-1β, and TNF-α and anti-inflammatory cytokine IL-10 *in vitro*. As indicated in Figure 2B, the levels of IL-6, TNF-α, and IL-1β were greatly increased, and the IL-10 was decreased in the LPS treatment group, whereas intervention with naringin can dose-dependently reverse this phenomenon. Therefore, naringin has an anti-inflammatory effect *in vitro*.

### 3.3 Naringin treatment decreased the ROS production during LPS-induced endometritis

**FIGURE 3:**
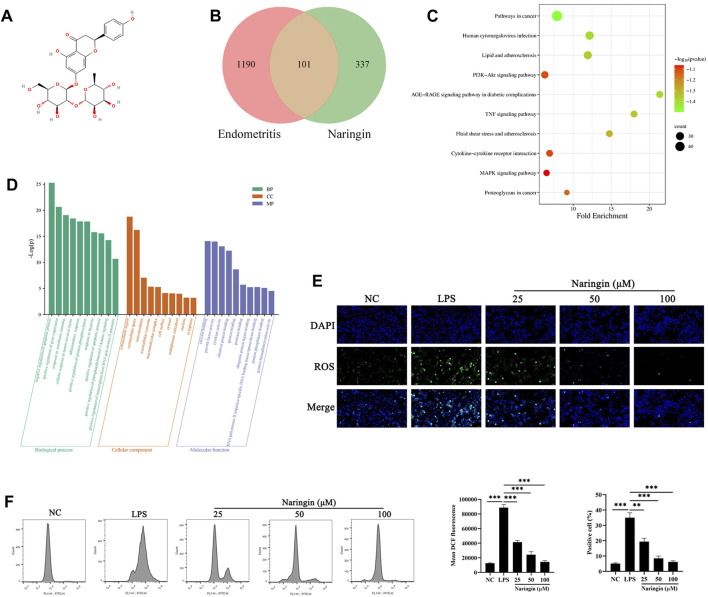
Network pharmacology analysis and ROS level of naringin. **(A)** Chemical structure of naringin. **(B)** Venn diagram of naringin target genes and inflammatory disease. **(C,D)** KEGG pathway enrichment analysis and GO enrichment analysis for key targets (top 10 are listed). The abscissa label represents the fold enrichment of the pathways. **(E,F)** The intracellular ROS levels were measured by staining with DCFH-DA and then determined by fluorescence microscopy (n = 3) and flow cytometry (bottom, n = 2). Scale bars, 50 μm. The data are presented as the mean ± SEM. Experiments were repeated n times with duplicate biological replicates. **p < 0.01; ***p < 0.001.

In order to explore the anti-inflammatory mechanism of naringin, we investigated the therapeutic targets involved in the naringin treatment of endometritis by network pharmacology. As shown in Figures 3A, B, we found that naringin has 101 putatively identified target genes associated with uterine inflammatory damage. We conducted GO annotation on these target genes, and the results showed that they are mainly related to the apoptotic process, inflammatory response to oxidative stress, and tumor response in BP enrichment analysis; extracellular, cell surface, mitochondrion, and ER in CC analysis; and enzyme binding, growth factor activity, cytokine activity, and protein binding in MF analysis (Figures 3C, D). The result of the KEGG pathway enrichment analysis indicated that target genes were significantly enriched in cancer pathways, the MAPK pathway, human cytomegalovirus infection, and the PI3K-AKT signaling pathway. Considering the results of network pharmacology analysis and previous studies indicating that naringin is an antioxidant, we speculate that naringin inhibits the development of endometritis by regulating ROS levels (Chen et al., 2015). Therefore, we measured whether naringin pretreatment could inhibit an LPS-triggered increase in ROS levels. As shown in Figures 3E, F, naringin inhibited the LPS-triggered oxidative stress in a dose-dependent manner.

### 3.4 Naringin alleviated the LPS-induced ERS in bEECs

**FIGURE 4:**
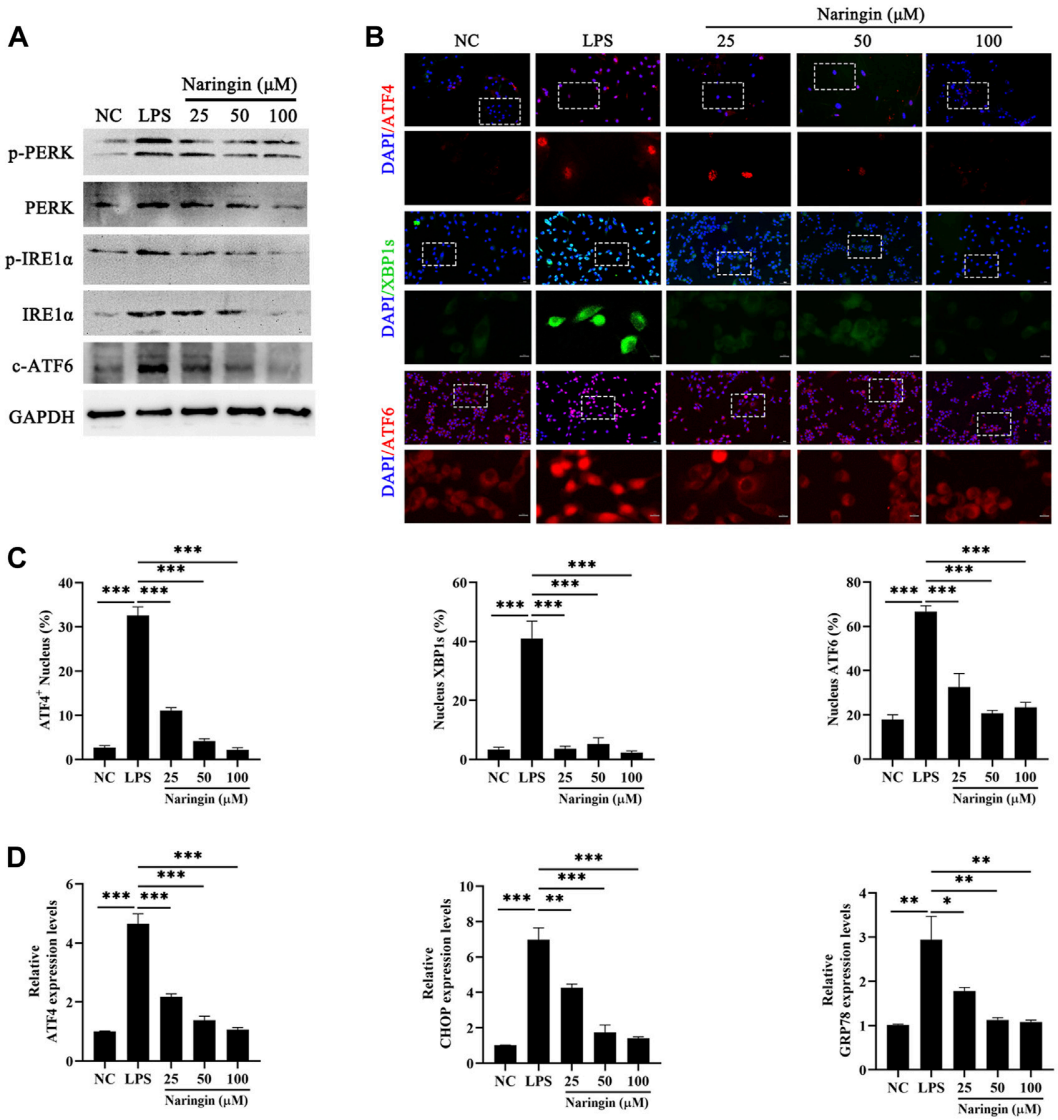
Effects of naringin on LPS-induced ERS. **(A)** The protein expressions of p-PERK, PERK, pIREα, IREα, and cleaved ATF6 were determined in triplicate by using Western blot analysis. n = 3. **(B,C)** A confocal image provided by immunofluorescence determined the expression levels of ATF4, XBP1, and ATF6 in bEECs. Scale bars: 50 μm and 10 μm (bottom, n = 2). **(D)** RT-qPCR analysis of ATF4, CHOP, and GRP78 mRNA expression normalized to the expression of GAPDH. n = 4. The data are presented as the mean ± SEM. Experiments were repeated n times with duplicate biological replicates. *p < 0.05; **p < 0.01; ***p < 0.001.

ERS is an inflammation marker that is involved in a wide range of disease processes (Grootjans et al., 2016), which usually can be activated by ROS abnormal homeostasis. Here, we measured the protein level of PERK, IRE1α, and ATF6, which are three ERS sensors. The results showed that naringin inhibited the LPS-stimulated increase in the phosphorylation of PERK and IRE1α, as well as the expression of ATF6, compared to the control group (Figure 4A and attached Supplementary Figure 3). We also evaluated the level of downstream UPR in ERS by naringin, including the core genes ATF6, ATF4, and XBP1s of the three classic UPR transcriptional activator pathways. Immunofluorescence results showed significant nuclear transfer in ATF6, ATF4, and XBP1s (Figures 4B, C). In addition, we found that ATF4, C/EBP homologous protein (CHOP), and GRP78 were inactivated after naringin treatment (Figure 4D). These data indicated that naringin inhibited ERS *in vitro*.

### 3.5 Naringin suppresses the autophagic flux by inhibiting the ERS in bEECs

**FIGURE 5:**
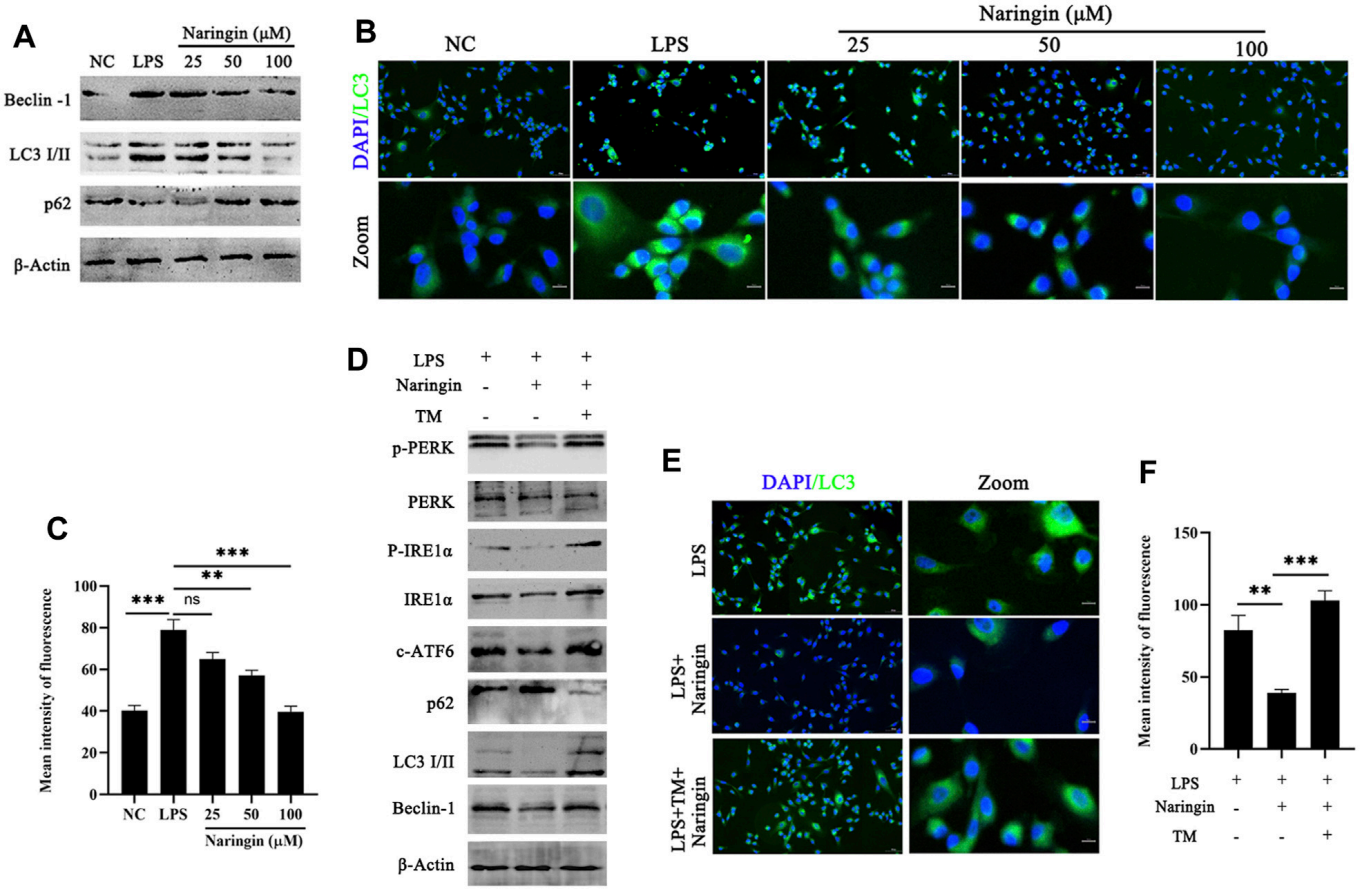
Naringin could inhibit autophagy induced by ERS. **(A)** The protein expressions of LC3, Beclin-1, and p62 were determined in triplicate by using Western blot analysis. n = 3. **(B,C)** Immunofluorescence staining showing the expression of LC3 in bEECs. n = 2. **(D)** Lysates of bEECs treated with LPS and then subjected to naringin (50 μM) and TM (5 ug/mL, 2 h prior to naringin treatment) for 24 h were analyzed for the presence of the indicated proteins. n = 3. **(E,F)** Immunofluorescence staining (left, n = 2) and quantitative analysis (right, n = 9) of the expression of LC3 on bEECs after the above treatment. The data are presented as the mean ± SEM. Experiments were repeated n times with duplicate biological replicates. **p < 0.01; ***p < 0.001.

Considering the UPR response pathway activated by ERS, the ATF6, IRE1α, and pERK pathways can control the quality of ER by regulating cell autophagy to complete cell self-rescue or apoptosis. Figure 4D indicates that the autophagy regulatory factors CHOP and XBP1 are inhibited by naringin, so we speculate that naringin exerts its anti-inflammatory effects through the ER autophagy system. Therefore, we evaluated the effect of naringin on autophagy activation of bEECs by Western blotting. Our results showed that with the gradual increase in the naringin concentration, the ratio of LC3-II to LC3-I and the Beclin-1 levels decreased, while the levels of p62 were the opposite, indicating that the naringin inhibits autophagy in a concentration-dependent manner (Figure 5A and attached Supplementary Figure 4A). Subsequently, we further evaluated the level of autophagy by immunostaining LC3. Our results showed that the fluorescent intensity of the naringin group was weaker than that of the LPS treatment control group (Figures 5B, C), indicating that the naringin indeed suppresses the autophagic flux. Interestingly, we treated bEECs with a combination of naringin and tunicamycin (TM, ERS inducer) and found that TM could significantly induce ERS and autophagy inhibited by naringin as determined by Western blotting (Figures 5D, E and attached Supplementary Figure 4B). Meanwhile, immunofluorescence staining results also showed that the naringin and tunicamycin treatment groups produced more autophagosomes, indicating that LPS-induced endoplasmic reticulum stress can significantly activate autophagy (Figures 5E, F).

### 3.6 Naringin inhibiting autophagy by activated PI3K/AKT pathway activation in bEECs

**FIGURE 6:**
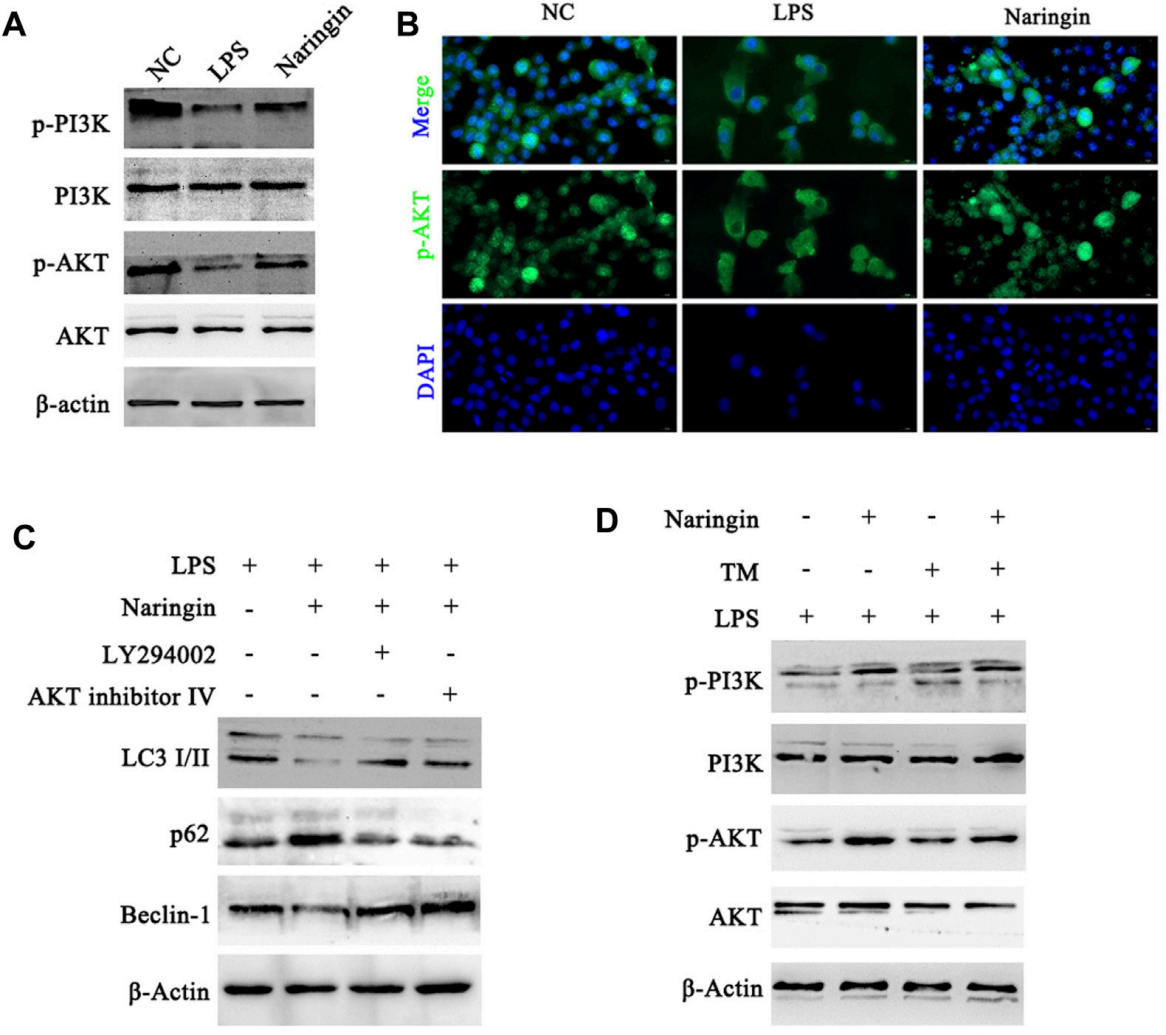
Naringin acts via the PI3K/AKT pathway to regulate autophagy in bEECs. **(A)** Total cell lysates of bEECs treated with LPS and/or naringin (50 μM) were prepared and subjected to immunoblot analysis with the indicated antibodies. n = 3 **(B)** Immunofluorescence staining showing the expression of p-AKT in bEECs after the above treatment. n = 2. **(C,D)** Lysates of bEECs treated with LPS and then subjected to naringin (50 μM) for 24 h were analyzed for the presence of the indicated proteins. LY294002 (10 μM), AKT inhibitors IV (2 μM), and TM (5 ug/mL) were administered 2 h prior to naringin treatment (left, n = 3). Experiments were repeated n times with duplicate biological replicates.

Next, the molecular mechanism of naringin that regulates autophagy to resistance to ERS was explored. The studies showed that the expression levels of p-PI3K and p-AKT in the naringin group were significantly increased compared with those of the control group (Figure 6A and attached Supplementary Figure 5A). The immunofluorescence results of p-AKT in bEECs also confirmed that naringin promoted the activation of the PI3K/AKT pathway (Figure 6B). Furthermore, we used LY294002 (PI3K inhibitor) and AKT inhibitor Ⅳ (AKT inhibitor) to treat with bEECs, and the autophagy markers were detected by Western blotting. LY294002 and AKT inhibitor Ⅳ reversed the effects of naringin on the LC3II/I ratio and the expression of Beclin-1 and p62 (Figure 6C and attached Supplementary Figure 5B). This indicates that naringin inhibits autophagy levels by activating the PI3K/AKT signaling pathway. In addition, we found that the ERS-induced TM can also inhibit the PI3K/AKT pathway, and naringin can partially reverse this effect (Figure 6D and attached Supplementary Figure 5C). These results indicated that the pharmacological mechanism of naringin’s anti-inflammatory effect is to weaken ERS and then suppress autophagy by inhibiting the PI3K/AKT pathway in bEECs.

### 3.7 Naringin administration decelerates autophagy in mice with endometritis and promotes the repair of inflammation injury

**FIGURE 7:**
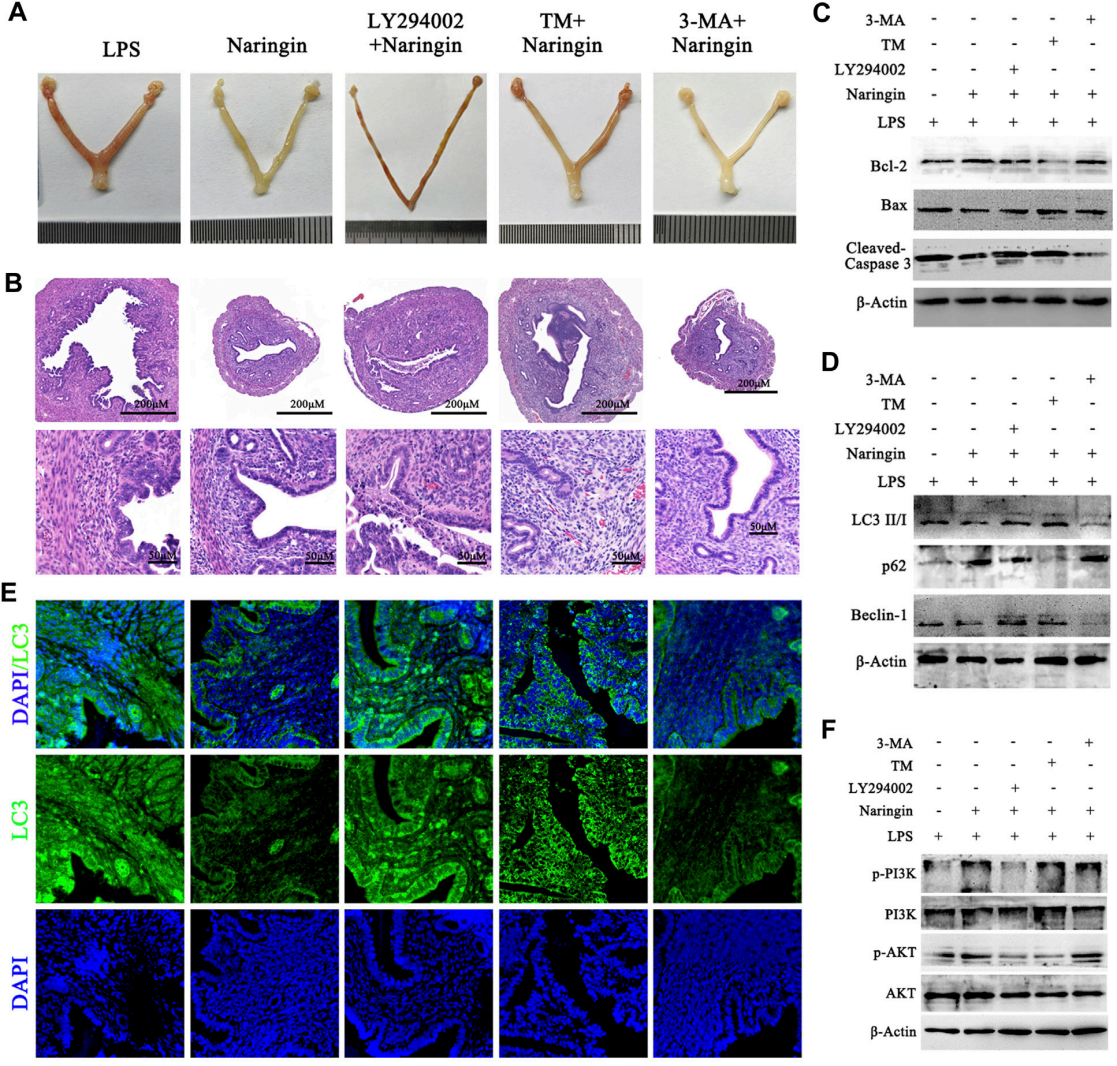
Naringin alleviates LPS-induced uterine injury via the ERS–PI3K/AKT–autophagy axis. Uterine tissues were collected from inflammatory-infected mice treated with naringin and 3-MA, LY294002, and TM for 24 h, with six mice in each group. **(A)** Representative whole uterus images. **(B)** Representative histopathological images of paraffin-embedded mouse uterine sections. Scale bar: 200 μm (top) and 50 μm (bottom, n = 3). **(C,D)** Total lysates from mouse endometrial tissue were prepared and subjected to immunoblot analysis with the indicated antibodies. n = 5. **(E)** Representative immunofluorescence images of LC3 in uterine sections. Scale bar: 20 μm, n = 2. **(F)** Total lysates from mouse endometrial tissue were prepared and subjected to immunoblot analysis with the indicated antibodies. n = 5. Experiments were repeated n times with duplicate biological replicates.

Finally, to further verify the correctness of the *in vitro* conclusions, 3-MA, LY294002, and TM were administered to mice in combination with naringin and LPS. The results indicated that LY294002 and TM treatment partly abolished the protective effects of naringin on uterine injury (Figures 7A, B) and cell apoptosis (Figure 7C and attached Supplementary Figure 6A). Furthermore, the level of autophagy was confirmed by immunoblotting for LC3 II, beclin-1, and p62 and by immunofluorescence for LC3 II. As expected, LY294002 and TM promoted autophagy and were inhibited by naringin, while the 3-MA inhibitor group intervened in the opposite direction (Figures 7D, E and attached Supplementary Figure 6B). Similarly, we also found that the activation of the PI3K/AKT pathway was consistent with *in vitro* experiments (Figure 7F; Supplementary Figure S6C). Overall, our study confirmed that naringin exerts anti-inflammatory effects by regulating the ERS–autophagy system through the PI3K/AKT pathway. It should be noted that whether *in vivo* or *in vitro*, blocking the PI3K/AKT pathway with LY294002 or activating ERS with TM cannot completely eliminate the effect of naringin on endometritis, which may be due to their inability to completely inhibit the PI3K/AKT pathway. However, it is more likely that naringin can play an anti-inflammatory role through a potential mechanism independent of the ERS-PI3K/AKT-autophagy system.

## 4 Discussion

Cow endometritis often causes repeated implantation failure and infertility, thus leading to serious economic losses for the modern dairy industry (Brodzki et al., 2014). At present, the clinical application of antibiotics in cow endometritis has achieved certain treatment effects, but new therapeutic strategies for endometritis are needed, as the problems of bacterial resistance and drug residues remain unresolved. In this study, we found that naringin, which is the main bioactive polyphenol in citrus fruits, alleviated inflammatory injury by inducing the ERS–autophagy system *in vivo* and *in vitro*. Mechanistically, we suggested that naringin decreased the production of ROS and then promoted the activation of the PI3K/AKT/mTOR pathway (*Graphical Abstract*).

The naringin focused in this study is a monomeric component widely present in nature, which can be extracted from fruits and vegetables such as citrus, grapefruit, green peel, and tomato (Chen et al., 2016; Dong et al., 2023). Due to its easy availability and close correlation with human food, researchers are curious about its pharmacological properties to determine its potential for patent medicine. As a kind of bioactive polyphenol, reports indicate that it has strong bioactivity in repairing bone defects, reducing blood lipids, and has anti-tumor, anti-inflammatory, and anti-atherosclerosis effects and can treat other diseases (Chen et al., 2015; Aihaiti et al., 2021; Raghu et al., 2023). In this study, we found that naringin can alleviate the structural and functional damage of the endometrium caused by LPS while accelerating the regression of inflammation by reducing LPS-induced secretion of inflammatory factors both *in vivo* and *in vitro*, exhibiting significant anti-inflammatory activity. This characteristic of naringin can be seen in studies on its relationship with colitis and pneumonia (Kim et al., 2018; Dong et al., 2021). It is worth noting that the molecular mechanism of naringin's anti-inflammatory effects is unclear in various types of inflammation, especially because the potential molecular mechanism of naringin in treating endometritis has not been reported.

Network pharmacology analysis is a new approach to analyzing the pharmacological properties and potential mechanisms of drugs based on networks of pharmaceutical active ingredients and targets (Nogales et al., 2022). First, we screened 101 therapeutic targets for the treatment of endometritis with naringin. Next, enrichment analyses of the GO and KEGG pathways were carried out, suggesting that these targets are enriched in multiple biological processes and pathways, such as those related to ER, inflammation, hypoxia, and the PI3K/AKT pathway. In the following experiments, we also confirmed that some enriched pathways are associated with naringin in the treatment of endometritis, as shown in Figures 4–6. Naringin can improve the increase in ROS levels induced by LPS in bEECs, which is consistent with previous studies suggesting that naringin can alleviate oxidative stress by directly eliminating free radicals and increasing the activity of endogenous antioxidant enzymes (Chen et al., 2015; Akamo et al., 2021). According to reports, naringin’s antioxidant capacity is comparable to that of probucol and lovastatin, which are commercial antioxidants (Jeon et al., 2001; Jeon et al., 2002).

Increasing evidence strongly suggests that ERS is involved in regulating the occurrence and development of various types of inflammation through a UPR-activated NF-κB pathway or by regulating the release of inflammatory factors (Marciniak et al., 2022; Mochida and Nakatogawa, 2022). In this study, we found that LPS activated endometrial ERS by disrupting ROS homeostasis, and naringin improved this phenomenon by inhibiting the expression of p-PERK, ATF6, and IRE1α. However, some studies have found that hypoxia also induces ERS, and the activation of the ERS-UPR pathway can promote cell survival (Kar et al., 2023). One explanation for this contradiction is that the three UPR pathways have a dual effect on cell fate, dependent on the selective pathways activated or the duration of ERS (You et al., 2021). In addition, the UPR pathway is not the only pathway by which the body responds to ERS.

Currently, it is widely recognized that UPR passes through the corresponding IRE1α, PERK, ATF6, and Ca^2+^ pathways to regulate the macro-autophagy that selectively targets the ER (Mochida and Nakatogawa, 2022). In this study, we found that naringin inhibited autophagy in a concentration-dependent manner, and this effect is weakened when using inducers to induce ERS. Next, we understand the molecular mechanism involved in autophagy in endometritis intervention with naringin. The results indicate that naringin suppressed autophagy by activating the PI3K/AKT pathway in bEECs. In fact, mammals, which have the largest number and non-conservation of ER-phagy receptors, have diverse mechanisms to regulate different modes of ER-phagy (González-Rodríguez et al., 2022). The relationship between ERS conditions (such as ROS) and autophagy has been well studied, and there is evidence that autophagy is activated under ERS conditions, which can further trigger ERS (Park et al., 2020; Kucińska et al., 2023). According to the observation of the markers of UPR and autophagy expression, ERS induced by LPS preceded the onset of autophagy.

## 5 Conclusion

Naringin was able to reverse the effects of LPS, which causes oxidative stress and ERS, by reducing the release of inflammatory cytokines and apoptosis in bEECs and mice. Additionally, naringin improves cell apoptosis caused by ROS and ERS mainly through the inhibition of autophagy on the PI3K/AKT axis. Collectively, these observations demonstrated that naringin could have a potential protective effect on LPS-induced endometritis, which also provides a novel approach to investigating the potential targets of traditional medicine.

## Data Availability

The original contributions presented in the study are included in the article/Supplementary Material, further inquiries can be directed to the corresponding authors.
