# Supplementary material for: Naringin attenuates inflammatory injury to the bovine endometrium by regulating the endoplasmic reticulum stress–PI3K/AKT–autophagy axis
Source: Front Pharmacol. 2024 Aug 21;15:1424511. doi: 10.3389/fphar.2024.1424511 (PMC11371590; doi:10.3389/fphar.2024.1424511)

Attached Figure 1

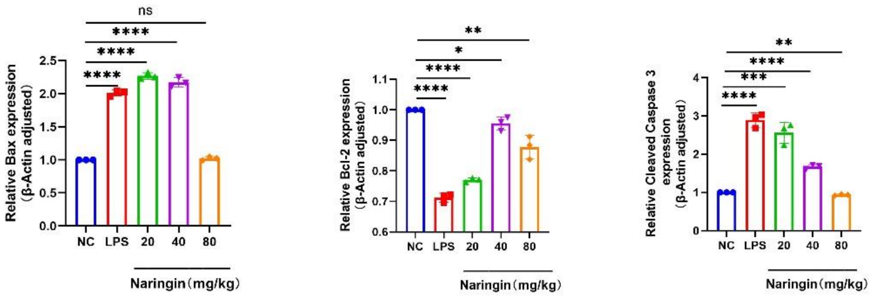

Attached Figure 2

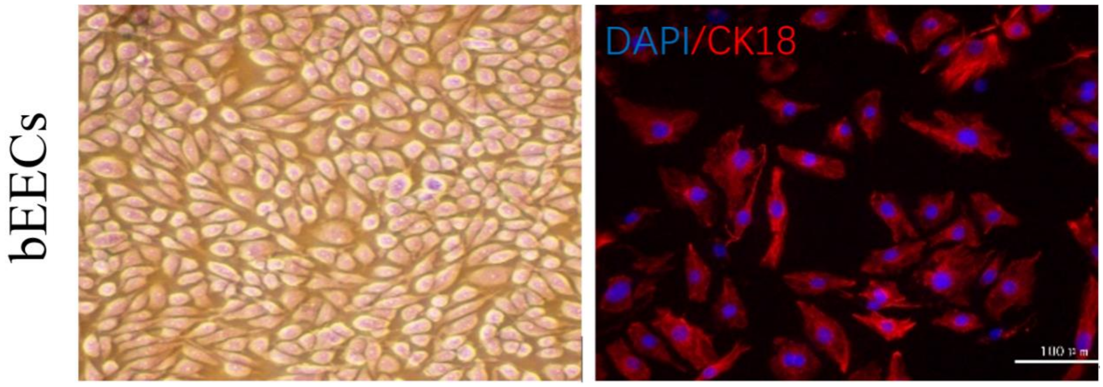

Attached Figure 3

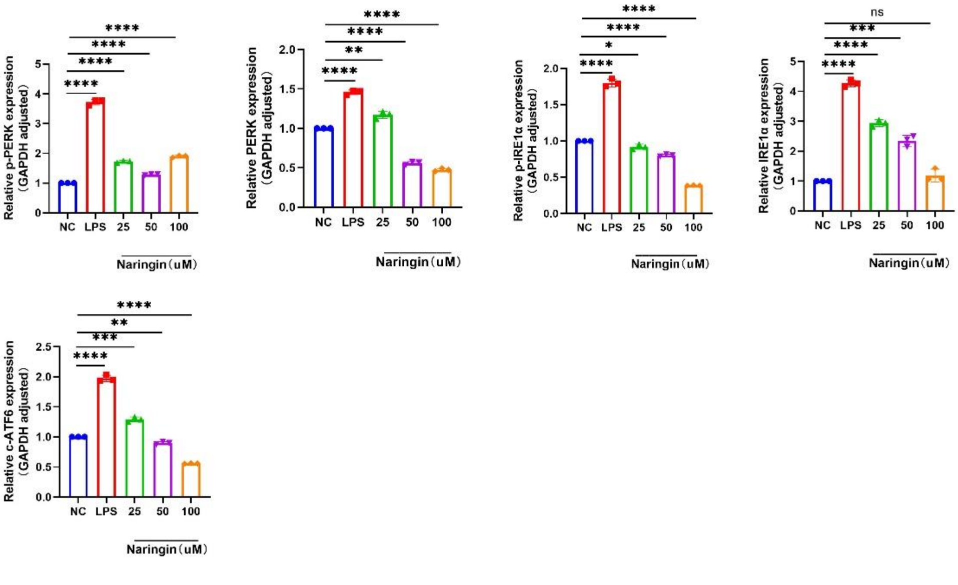

Attached Figure 4

A

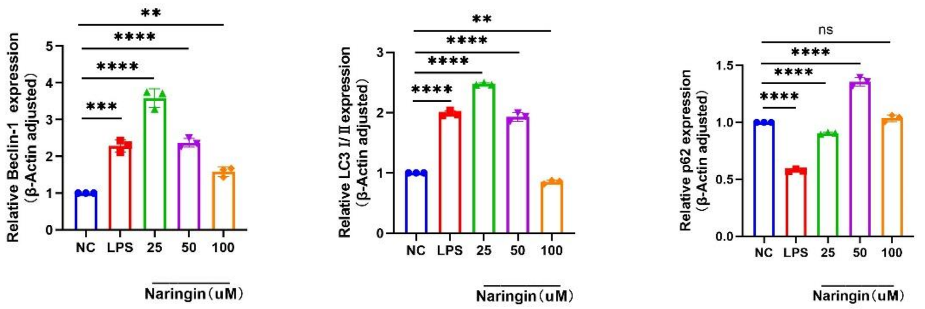

B

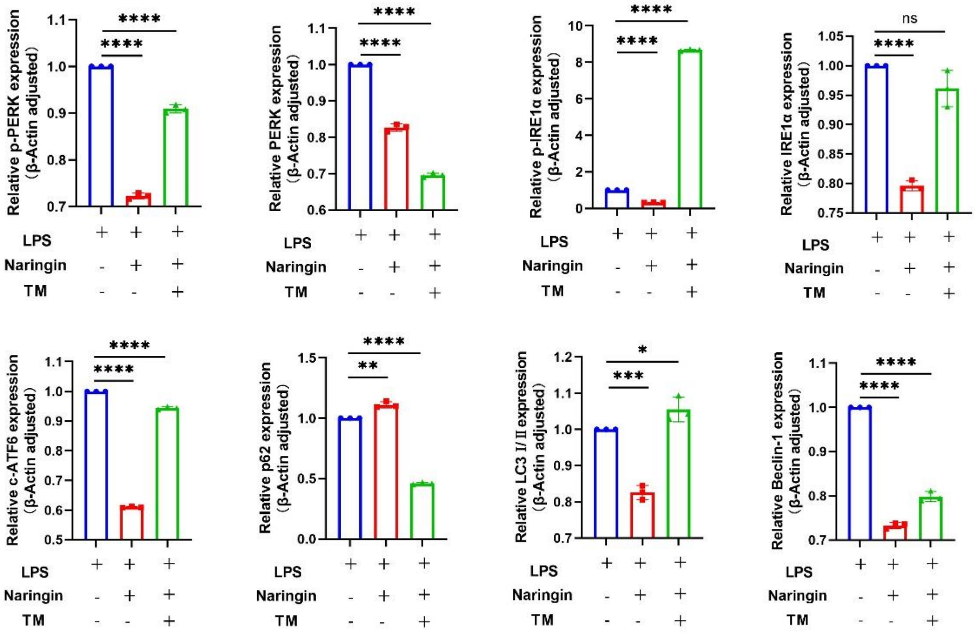

Attached Figure 5

A

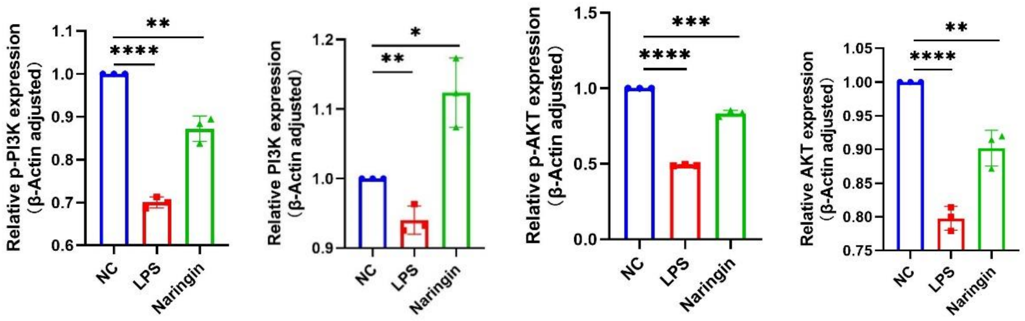

B

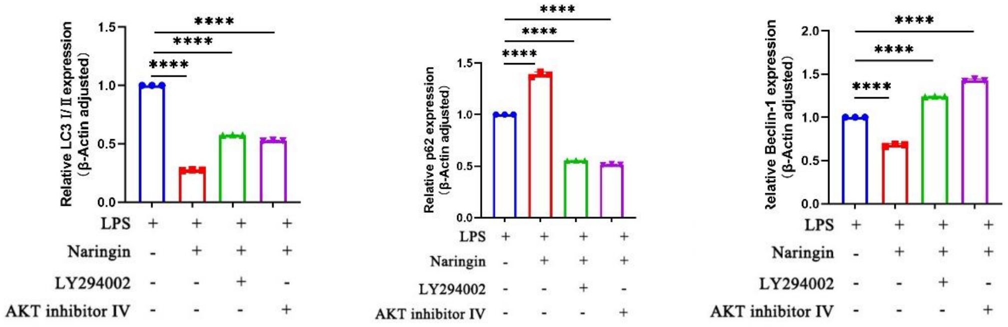

C

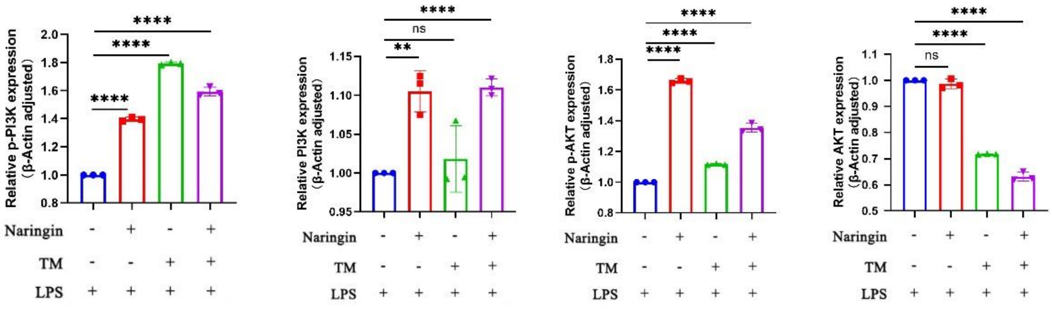

Attached Figure 6

A

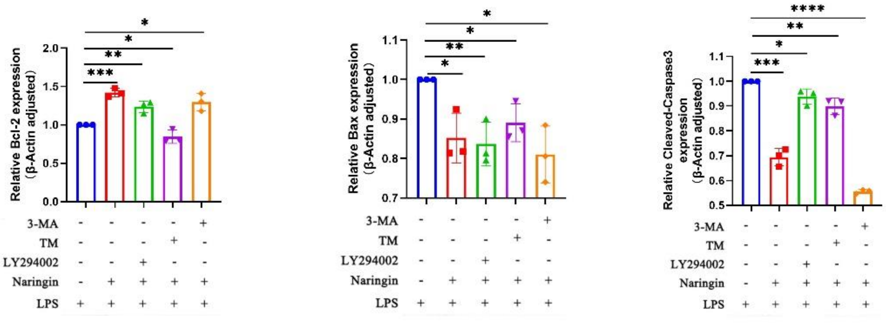

B

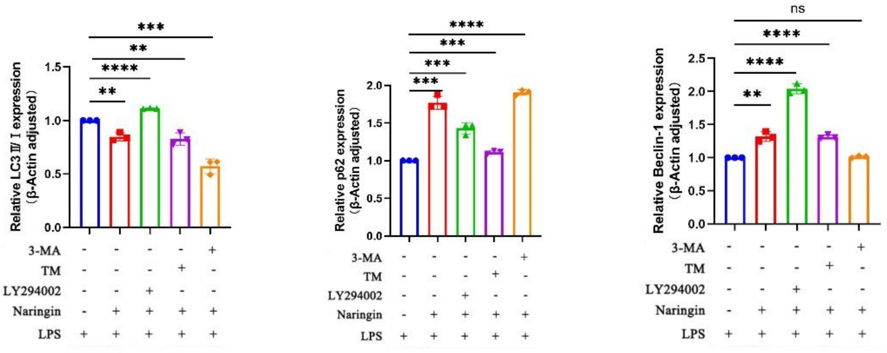

C

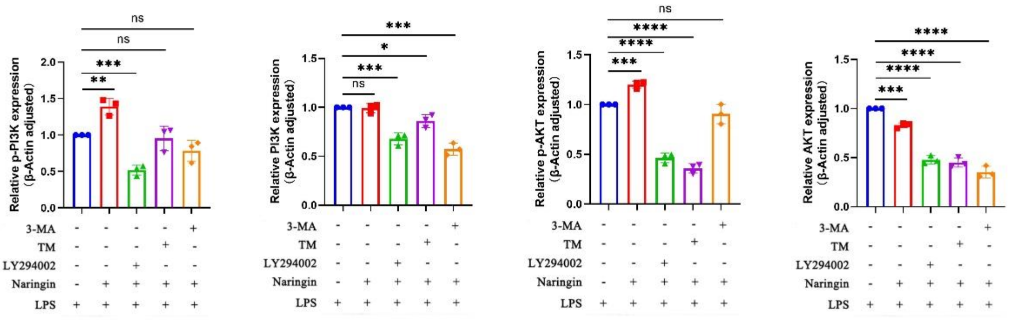

Fig.1D

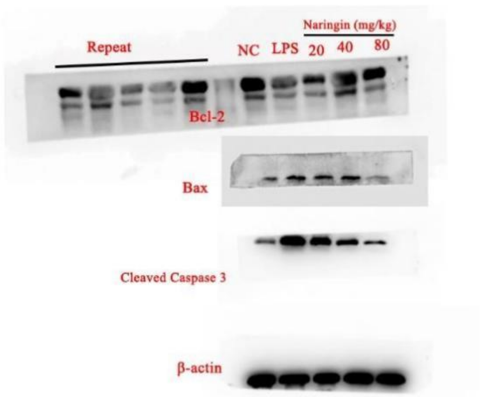

Fig.4A

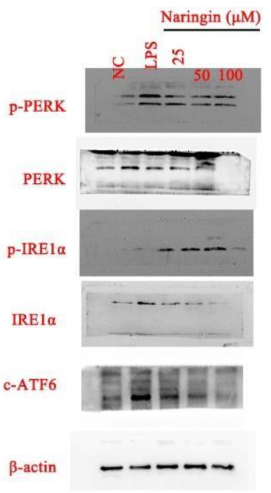

Fig.5A

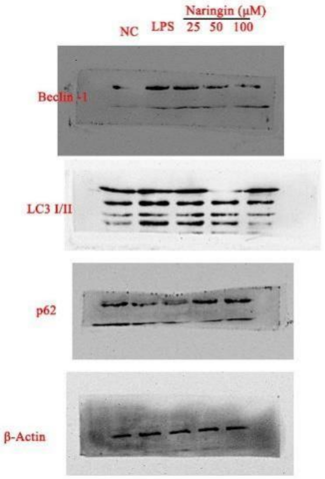

Fig.5C

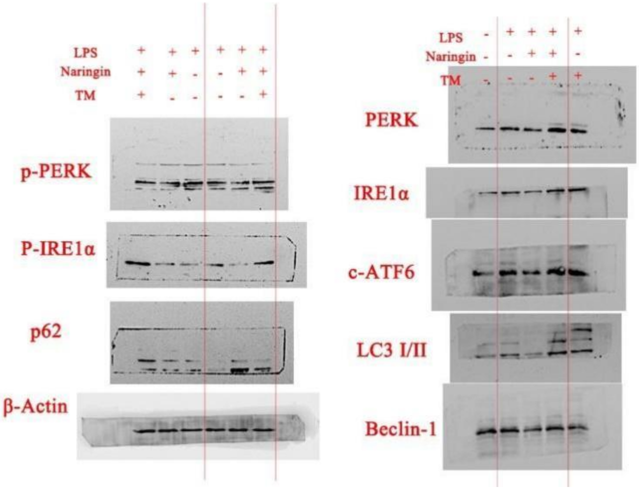

Fig.6A

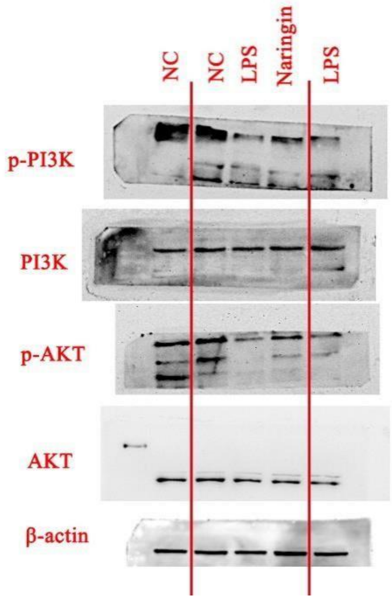

Fig.6C

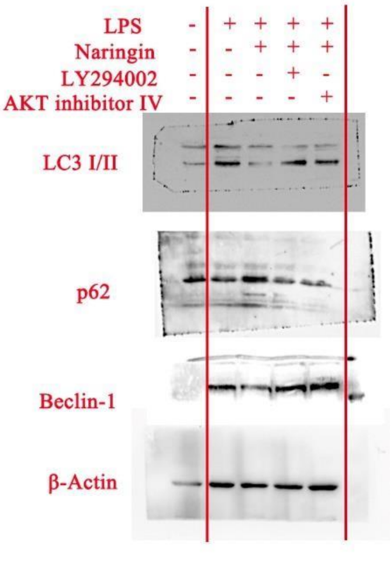

Fig.6D

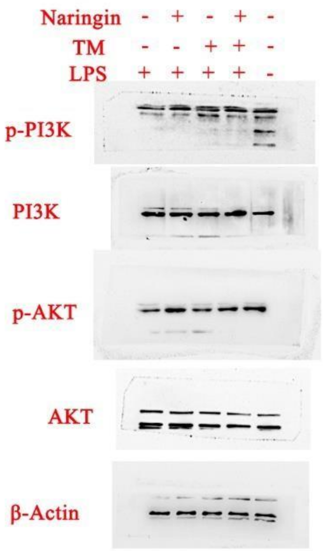

Fig.7B

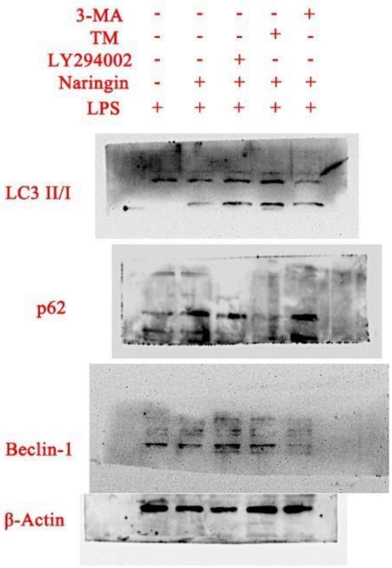

Fig.7C

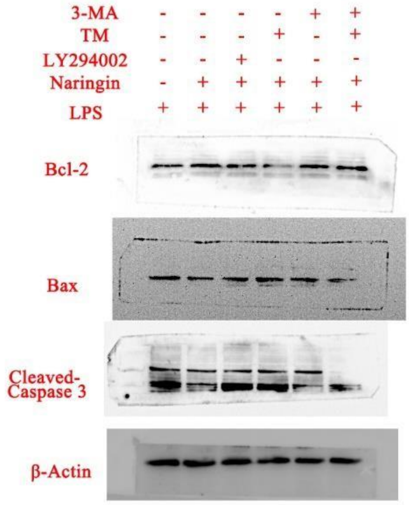

Fig.7E

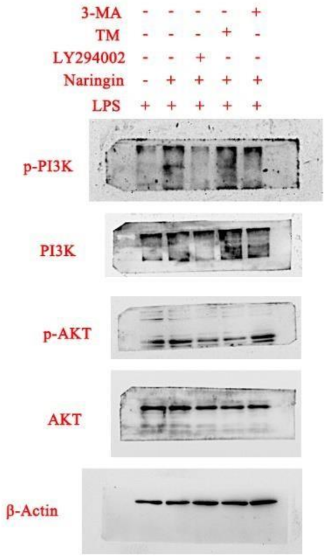

Supplement: Supplementary file 1 [file DataSheet1.pdf]
